# Optimal Resource Provisioning and Task Offloading for Network-Aware and Federated Edge Computing

**DOI:** 10.3390/s23229200

**Published:** 2023-11-15

**Authors:** Avilia Kusumaputeri Nugroho, Shigeo Shioda, Taewoon Kim

**Affiliations:** 1 School of Computer Science and Engineering, Pusan National University, Busan 46241, Republic of Korea; avilia22@pusan.ac.kr; 2 Graduate School of Engineering, Chiba University, Inage-ku, Chiba 263-8522, Japan; shioda@faculty.chiba-u.jp

**Keywords:** mobile edge computing, task offloading, optimal association, vertical scaling, horizontal scaling

## Abstract

Compared to cloud computing, mobile edge computing (MEC) is a promising solution for delay-sensitive applications due to its proximity to end users. Because of its ability to offload resource-intensive tasks to nearby edge servers, MEC allows a diverse range of compute- and storage-intensive applications to operate on resource-constrained devices. The optimal utilization of MEC can lead to enhanced responsiveness and quality of service, but it requires careful design from the perspective of user-base station association, virtualized resource provisioning, and task distribution. Also, considering the limited exploration of the federation concept in the existing literature, its impacts on the allocation and management of resources still remain not widely recognized. In this paper, we study the network and MEC resource scheduling problem, where some edge servers are federated, limiting resource expansion within the same federations. The integration of network and MEC is crucial, emphasizing the necessity of a joint approach. In this work, we present NAFEOS, a proposed solution formulated as a two-stage algorithm that can effectively integrate association optimization with vertical and horizontal scaling. The Stage-1 problem optimizes the user-base station association and federation assignment so that the edge servers can be utilized in a balanced manner. The following Stage-2 dynamically schedules both vertical and horizontal scaling so that the fluctuating task-offloading demands from users are fulfilled. The extensive evaluations and comparison results show that the proposed approach can effectively achieve optimal resource utilization.

## 1. Introduction

In recent years, the growing number of delay-sensitive applications operating on resource-constrained devices has presented major challenges to the efficient execution of these applications. In particular, the emergence of the Internet of Things (IoT) has highlighted the need for efficient and responsive computing solutions [1]. To address these challenges, mobile edge computing (MEC) has emerged, which is regarded as a distributed version of cloud computing. MEC has been widely studied in depth [2,3,4,5] as a promising solution to offloading resource-intensive tasks to edge servers located in the vicinity of the user devices [6,7,8]. In particular, the common goals are reducing network latency [9], enhancing responsiveness [10], and improving quality of service (QoS) [11]. By utilizing the rich computation and storage resources of edge servers, MEC can accelerate the performance of applications running on user devices. In addition, it conserves the limited resources of end-devices, thereby preserving their operational lifetime.

However, the effectiveness of MEC depends on a well-designed resource scheduling strategy that makes optimum utilization of the resources that are related to each other. In particular, the optimal utilization of MEC required two key aspects to be addressed: the association of users with base stations (BS), the provisioning of virtualized resources on edge servers (ES), and the dynamic distribution of tasks across edge servers. Achieving an optimal balance among these factors is of significance, as it can result in reduced response time and battery consumption at user devices. User-edge server association optimization [12] is a fundamental aspect of MEC optimization that ensures the balanced utilization of edge servers. For example, the balanced distribution of users across edge servers can prevent over- or under-provisioning problems. This not only enhances the QoS for individual users but also maximizes the efficiency of the MEC system as a whole. Achieving such balanced user-edge server associations requires careful consideration of factors such as workload distribution [13].

Due to its importance, various MEC optimization approaches have been explored [14,15,16,17]. However, the optimal use of MEC involves several challenges from various aspects. Many optimization methodologies currently in use have a limited scope, focusing on individual challenges within the MEC architecture. These optimization efforts frequently concentrate on individual elements such as edge server resource allocation, task-offloading strategies, and user-server assignments. Although these approaches provide valuable insights and opportunities for performance enhancement, their limits arise from their constrained scope. They often overlook considering comprehensive factors such as network resource assignments, vertical/horizontal resource provisioning, and integrated task offloading. These limitations emphasize the need for an approach that integrates all of these factors.

An optimal MEC approach should consider a wider range of factors, from the capabilities of individual edge servers to the network as well as the dynamic requirements of users and applications. In general, an edge server is located inside or connected via a direct link to a base station [18], and thus the association between user and base station can also determine the association between user and edge server. One key strength of edge computing is the capability of carrying out scaling in a dynamic manner. Optimized scaling can be performed either vertically or horizontally. The previous studies optimized them either independently instead of considering them together as an integrated approach to resource management. While several studies have explored the benefits of vertical scaling (VS) [19,20], horizontal scaling (HS) strategies have also garnered attention for their potential [21,22]. VS involves adjusting the capacity of individual edge servers by reallocating computational resources such as CPU, memory, and storage to handle varying workloads. HS, on the other hand, focuses on adding or removing edge servers to adapt to changing demands and maintain optimal system performance.

Despite the study conducted on each scaling approach, there is still a need for further research to enable both VS and HS. Expanding on this concept, our research emphasizes the potential of enabling both VS and HS in order to achieve enhanced resource utilization and QoS. By combining both scaling approaches, we create a dynamic environment in which not only the capacity of individual edge servers but also the number of edge servers is adjusted to handle varying workloads. This approach effectively utilizes the benefits of optimization, scaling, and responsiveness. Furthermore, joint consideration is also a crucial aspect of enhancing system efficiency. Network-side optimization ensures the efficient functioning of user-base station associations, while MEC optimization enhances the performance of resource allocation (both VS and HS) and task offloading. Although these aspects are often viewed as distinct notions, their integration has the potential to significantly reduce response times.

To further enhance the optimal MEC strategy, it should consider a wide range of factors, including the capabilities of individual edge servers, the effects of network resource scheduling, and dynamic user and application requirements. In addition, we consider the single-provider system of MEC, where a single service provider operates its own edge servers, each with different features and constraints. An organization or business can lease one or more edge servers, which can be exclusively used by the corresponding service subscribers. In such a scenario, a fully centralized optimization strategy is feasible for the single provider with its own regulations and requirements.

The concept of edge server federation represents a plausible use case, necessitating an examination of how the introduction of federation impacts dynamics and resource allocation. This prompts questions about resource management across federations, the feasibility of high-speed communication between federations, and the implications for user assignments and task offloading in a federated setup. Notably, the dynamics of resource allocation, load balancing, and task management may differ significantly when compared to a non-federated edge server environment.

This organizational structure of edge servers, organized into distinct federations, serves different purposes, whether for security reasons or to accommodate various organizations. The significance of this organizational structure increases in contexts with single providers. In instances where an individual provider operates multiple federations of edge servers, each of which caters to a distinct function, centralized optimization may be more applicable within each distinct federation. One additional factor to point out is that when edge servers are operated by different operators or when security is of important concern [23], horizontal scaling, or even the migrations [24] can be allowed only within a federation of edge servers.

Given the aforementioned considerations, our research methodology aims to provide an integrated optimization approach that effectively incorporates edge server provisioning by merging the principles of both VS and HS and task offloading assignment, alongside network resource scheduling. The comprehensive methodology holds potential for finding novel approaches to enhance performance and optimize resource utilization in a federated edge computing environment. The contributions of this paper are summarized as follows:

- In contrast to previous optimization approaches that focused on each individual component of the MEC architecture, we propose a comprehensive optimal approach that optimizes a chain of components in the MEC as well as network resources. In this study, the proposed approach optimizes BS-user association, federated-user assignment, resource provisioning, and task offloading.
- We propose a *federated* edge server-based MEC architecture, where a user assigned to a particular federation can utilize only the edge servers in the same federation. This is practical and essential when some edge servers are operated by different service provider, or when some edge servers are owned by a third-party organization that is not trustworthy.
- In contrast to the previous MEC optimization approaches that focused on either VS or HS for provisioning virtualized resources, we propose to enable both to further enhance resource utilization and users’ QoS.
- We refer to our proposed approach as NAFEOS, which stands for Network-Aware Federated Edge Computing Optimal Scheduling. The NAFEOS approach presented in this study is formulated as a two-stage algorithm, considering the execution interval and complexity. The Stage-1 problem, which runs at long intervals, includes binary variables, resulting in relatively higher complexity. However, due to the efficient algorithms, such as branch-and-bound and branch-and-cut, leveraged in the computer solver we used in this study, the mixed integer (binary) linear program we propose in this study can be computed efficiently. On the other hand, the Stage-2 problem that iteratively optimizes both ES resources and task offloading at short intervals is formulated as linear programming so that it can run at low complexity. The proposed problem formulation aims to minimize battery consumption and service delay from the users’ perspective. At the same time, it maximizes the fair load distribution among federations by having a multi-objective optimization solution.
- We have carried out extensive evaluations and validated the effectiveness of the proposed optimal approach. Also, we have performed a comparison study with the common approaches. To do so, we implemented the proposed method along with its variants.

The remainder of this paper is structured as follows: Section 2 summarizes the related studies. The following Section 3 presents the proposed two-staged approach that optimizes network and edge server resources jointly. Evaluation, validation and analysis of the proposed solution are carried out in Section 4, and Section 5 concludes this paper.

## 2. Related Work

This section provides a comprehensive review of MEC and resource management literature. Prior studies have explored different aspects of MEC, often focusing on specific resource management components. User-BS associations have been an important part of MEC research. In line with our research, Wang et al. [25] proposed an optimization method for the association between users and base stations in MEC. This study prioritizes minimizing system delay and emphasizes efficient user-BS associations for enhancing QoS. The work, however, is limited in that it does not consider the scaling of virtualized resources, which is an essential factor in the optimal use of MEC.

In the context of user-edge server association in the context of MEC, Dai et al. [26] proposed a computational offloading framework that integrates both compute offloading and user-edge server association in a two-tier architecture. Tang et al. [27] introduced a task offloading approach that enhances the effectiveness of joint optimization techniques. The primary area of their research centers around optimizing tasks at the individual level. Despite their noteworthy findings related to the method of task offloading, the scope of this study was constrained to a single mobile device and a single mobile edge server. In addition, Bi et al. [28] proposed an integrated strategy for the joint optimization of computation-offloading decisions in MEC systems. Our method goes further by emphasizing the need for associations to enable low-latency communication within ES federations, facilitated by high-speed networks, all while considering federation-specific resource availability.

Edge server resource provisioning within MEC has also been studied. In [29], the study delved into MEC resource management within the Internet of Things (IoT) context, with a primary focus on optimizing resource efficiency and minimizing network costs. In other studies, ref. [30] proposed a comprehensive strategy concerning computing power allocation and efficient traffic scheduling for edge service provisioning. Furthermore, ref. [31] introduced the concept of resource provisioning in edge computing, with a special emphasis on applications demanding low-latency performance.

The exploration of federated edge computing has been conducted in various domains in several previous works [32,33,34]. Hussain et al. [32], introduced a federated edge computing approach for disaster management in remote smart oil fields, emphasizing resource allocation and load balancing for smart oil fields’ robustness. Chi et al. [33] proposed DEEP-NET, a fully decentralized on-demand MEC-SC peer-offloading network that emphasizes QoS-aware load balancing, improved latency, and the protection of service providers’ privacy. This approach leverages a federated gradient descent-based algorithm that operates in a fully decentralized manner. Karakoç et al. [34] proposed a Federated Edge Network Utility Maximization (FEdg-NUM) architecture, centered around clients with private utilities and communication within a peer-to-peer network of edge servers. While the aforementioned studies investigate federated edge computing across diverse domains, our research concentrates on network-aware resource provisioning and task offloading, encompassing a wider spectrum of edge servers and applications. Our work offers a comprehensive approach to resource optimization within the context of MEC, recognizing the significance of both MEC federating concepts and the federated edge computing paradigm.

In the optimization of MEC, the user-edge server association and the exploration of both VS and HS strategies have been studied as well. Regarding VS and HS, a cluster of notable research papers has been highlighted [35,36,37,38]. In [35], an innovative elastic edge cloud resource management model is proposed, which effectively combines the VS capability with HS. Expanding on this concept, ref. [36] proposes an adaptive auto-scaling technique for delay-sensitive serverless services. This method employs a complex combination of VS and HS that are intelligently tailored to the specific resource profiles of the services. Daraje et al. [37] presented a novel hybrid resource scaling strategy that stands out in the context of cloud computing. This method combines the capabilities of VS and HS in an effort to optimize resource utilization. However, it is notable that none of these approaches address the integration of optimal user-BS/ES association and dynamic task distribution comprehensively. Maia et al. [38] addressed the critical issue of optimizing the location of scalable Internet of Things (IoT) services within the domain of edge computing. Their research examines both VS and HS, highlighting the importance of service deployment and scaling in edge computing.

Compared to the studies discussed earlier, our work introduces a novel joint optimization framework for the chain of resources addressed above. This integrated methodology provides the flexibility to process the user’s offloading requests at either edge servers, the cloud, or even at the user’s device, thus enhancing the ES-cloud offloading system’s efficiency. Our work stands out for its capacity to handle these interdependent decisions collectively rather than separately. This work enables both VS and HS to further enhance the system’s performance and users QoS. Considering the notion of edge server federations raises the practicality of the proposed approach. The optimization of user-BS association, which is the network-side resource, also plays an important role, especially in edge computing with mobile devices, and thus it cannot be excluded from the task of MEC optimization. Overall, the comprehensive optimization for MEC proposed in this work results in optimized resource utilization and enhanced performance within the MEC ecosystem.

## 3. Proposed Idea

In this section, we illustrate the proposed system architecture, and then introduce the proposed optimal MEC management scheme.

### 3.1. Proposed System Architecture

**Figure 1:**
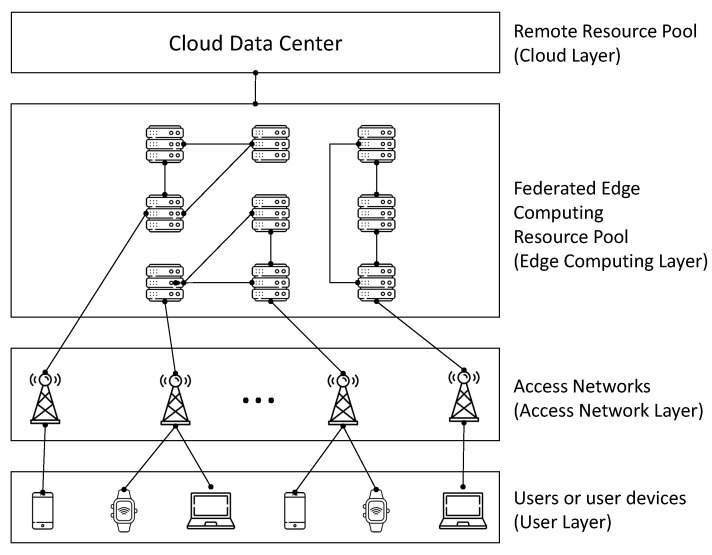
Overall system architecture consisting of four layers.

Figure 1 shows the overall system architecture we propose. The users (or user devices) at the bottom layer can connect to the network via access networks. Base stations (BS) at the access network can associate with users so that their offloading requests can be redirected to either ES(s) or the cloud. Each BS can associate and communicate with up to a certain number of users simultaneously, which is defined by the number of available orthogonal channels, $N_{CH}$. On top of the access network layer, ESs are deployed in federations. ESs belonging to the same federation can efficiently communicate with each other at low latency by being connected via a high-speed network. Such a system, for example, can be implemented by a software-defined network [39]. There is a one-to-one mapping between ES and BS, meaning that associating with a BS automatically determines which federation to belong to. To put it another way, to belong to a particular federation, a user should be associated with the BS that is connected to an ES in the federation. At the topmost layer is the cloud data center, which has enough computing resources, whereas the others, i.e., user devices and ESs, are resource-limited.

Offloading can be carried out in three different ways: self-offloading, offloading to ES(s), and offloading to the cloud. Users with enough computing resources on their devices can process their requests by themselves. Also, the user’s request can be offloaded to one or more ESs or the cloud. If HS is supported, a user can offload its request to multiple ESs in the same federation. Finally, excessive amount of requests can be offloaded to the cloud. The delay between a user and BS is very small compared to the rest of the delays we consider in this paper. To be specific, it can be computed by dividing the BS-user distance, e.g., a few hundreds of meters, divided by the speed of light which is normally 3 × 10${}^{8}$. The delay between ESs in the same federation is assumed to be short for being connected with each other by high-speed links. However, the links between ESs and the cloud are of large delay due to the large distance between ESs and the cloud. Users requests can be partitioned into fractional portions, and they can be processed in a distributed manner, possibly in different layers as well.

### 3.2. Assumptions

In this work, we make the following assumptions. The number of federation of ES, $N_{G}$, is known in advance, along with which ES belongs to which federation. Such a relation is abstracted by the federation indicator matrix $I_{grp}$$\in {\left\{0,1\right\}}^{N_{G}\times N_{S}}$, where $N_{S}$, the number of ESs or BSs in the system, is known in advance. In the matrix, if the *g*-th row and *s*-th column are one, ES *s* belongs to federation *g*. Users are assumed to be stationary with their locations known. Given the locations of BSs, the accessibility matrix $I_{acc}\in {\left\{0,1\right\}}^{N_{S}\times N_{U}}$ is constructed to indicate which user *u* can access (or within the transmission coverage of) which BS *s*, where $N_{U}$ is the known number of users in the system. In the matrix, if the *s*-th row and *u*-th column are one, user *u* can access BS *s*. A user can associate with a BS only when the user can access the BS. It is assumed that the average amount of task offloading requests for each user per time unit is known by using the historic logs, and denoted by r $\in {\left[0,1\right]}^{N_{U}\times 1}$. The computing resource budget available at users’ devices, ESs and the cloud is denoted by $c_{user}\in R_{++}^{N_{U}}$, $c_{es}\in R_{++}^{N_{S}}$ and $c_{cloud}$, respectively, where $c_{cloud}\in R_{++}$ is assumed to be a large number and $R_{++}$ indicates a strongly positive real number.

### 3.3. Proposed Optimal MEC Management Method

In this work, we propose a joint optimization of user-BS association, ES provisioning via VS and HS, and task distribution so that users’ requests can be processed efficiently. In contrast to the previous works focusing on each issue separately, we argue that such a chain of decisions should be considered at the same time to maximize the utilization of the ES-cloud offloading system. This is because one decision in a prior step can affect the following steps. Also, joint orchestration of VS and HS can further enhance the quality of service (QoS) of users as well as the resource utilization of the computing units (i.e., ES and cloud).

The proposed system consists of multiple layers as aforementioned, and there are multiple federations of ESs that are operated by different service providers. Associating a user with a BS leads to establishing a membership relation between the user and a federation as well. Thus, ill-considered association can yield undesired outcomes such as a certain federation being over-populated. Since a user can access the resource only within the same federation, it is not a desired situation. Also, BS can associate with up to a limited number of users, and thus an intelligent method for making an association and establishing a membership relation is required.

VS on ES can increase or decrease the allocated resources for the user’s request, but due to the limited resources available on each ES, it may not suffice to fulfill the user’s task-offloading demand. In such a case, allocating additional resources to other ES(s) is essential, called HS. Joint consideration of VS and HS can satisfy the user’s QoS, especially when the user’s demand is high or a certain ES is assigned to multiple users. Although the cloud resource pool is large enough, due to the increased delay when communicating with a remote cloud data center, it is desired to utilize as many ES resources as possible.

Considering the possible dynamic adjustment of ES resource allocation within a single ES or multiple ESs in the same federation, it is better to maintain enough amount of available, unused resources in each federation of ESs to be prepared for the possibility of upcoming offloading demand increase. In this paper, an effective load-balancing scheme among federations is proposed so that federations of ESs can process similar amounts of tasks and to secure enough amount of available resources therein.

Also, it is desired to use fewer resources on users’ devices since they are battery-limited. The objective of the proposed method is to achieve load balancing among federations of ES, to maximize the lifetime of users devices by minimizing the amount of tasks processed locally at the users’ devices, and to minimize the response time by minimizing the amount of tasks processed at the remote cloud data center.

**Figure 2:**
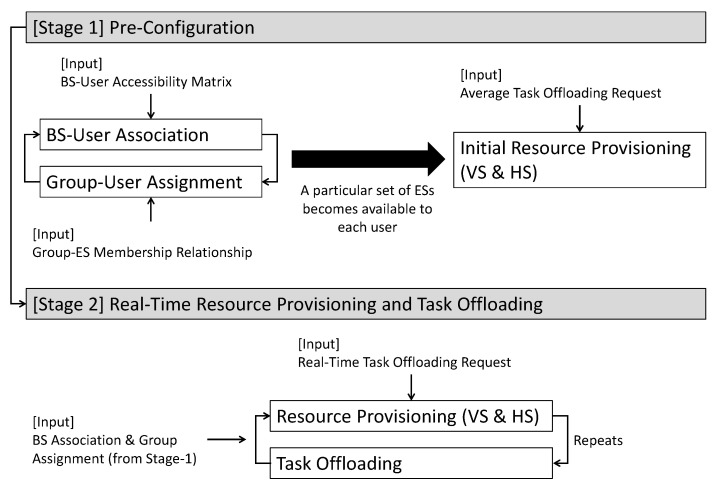
Overall flow of the NAFEOS method consisting of two stages.

The NAFEOS approach consists of two stages as shown in Figure 2: Stage-1: pre-configuration and Stage-2: real-time resource provisioning and task offloading. Stage-1 determines BS-user association and federated-user assignment. To make optimal decisions in the stage, Stage-1 also optimizes the resource provisioning and task offloading based on the average offloading requests from users. Once Stage-1 yields an optimal decision, the following Stage-2 iterates to make real-time optimal decisions regarding resource provisioning and task offloading upon receiving real-time task-offloading demand.

The Stage-1 optimization problem in NAFEOS can be formally presented as follows. The objective (1) is to minimize the three terms with the given weights α, β, and 1−α−β, where α+β≤1. The two non-negative design parameters do not exceed the value of 1, i.e., α,β∈[0,1], and the three strongly positive denominators $s_{1}$, $s_{2}$, and $s_{3}$ are used to scale the corresponding terms within the same range [0,1].

(1) $$\min_{\begin{array}{c} A,G,Y,\\ x_{user},X_{es},\\ x_{cloud},b \end{array}}\frac{\alpha }{s_{1}}b+\frac{\beta }{s_{2}}1_{1\cdot N_{U}}\times x_{cloud}+\frac{1-\alpha -\beta }{s_{3}}1_{1\times N_{U}}\cdot x_{user}$$

The first term in (1) minimizes *b* with which the offloaded load among federations can be balanced due to the constraint (15) to be addressed shortly. To be specific, the value of *b* is used to set the upper bound of the load across all federations and thus, minimizing *b* yields a fair load distribution. The second term minimizes the amount of task offloaded to the cloud (i.e., $x_{cloud}\in {\left[0,1\right]}^{N_{U}\times 1}$), where the *u*-th element in $x_{cloud}$ corresponds to the amount of user *u*’s load offloaded to the cloud. The main purpose of the second term is to reduce the response time since the large distance between the user and the cloud yields a large network delay. The third term minimizes the amount of self-offloading $x_{user}\in {\left[0,1\right]}^{N_{U}\times 1}$ (i.e., processing on the device itself), where the *u*-th element in $x_{user}$ determines the amount of self-offloading for user *u*. This term plays an important role in reducing the power consumption of the user device and prolonging its lifetime. The $1_{N\times 1}$ and $1_{1\times N}$ used in the objective function are a column and row vector of *N* number ones, respectively.

By minimizing the objective function, load balancing among federations can be guaranteed while both the service delay and battery consumption for users are minimized. To achieve the goal under practical considerations, we have defined the following constraints. The BS-user association decision is binary as shown below, called (2). The (s,u)-th element in A determines whether the BS *s* accepts the association request from user *u* or not by having the value be 1 or 0, respectively.

(2) $$A\in {\left\{0,1\right\}}^{N_{S}\times N_{U}}$$

In practice, the BS-user association can be made only when the user is placed within the transmission range of a BS. The following constraint (3) places an element-wise less-than or equal condition between A and $I_{acc}$ with the ⪯ operator. The binary constant of the (s,u)-th element in $I_{acc}$ corresponds to whether the user *u* can receive the pilot signal from the BS *s* or not by having the value of 1 or 0, respectively. Thus, the constraint (3) facilitates the BS-user association only when both can communicate with each other.

(3) $$A⪯I_{acc}$$

In this work, we assume a single antenna device for users and thus, each user can associate with a single BS at a time by the following constraint (4), where ≃ is the element-wise equal operator.

(4) $$A^{T}\times 1_{N_{S}\times 1}≃1_{N_{U}\times 1}$$

Each BS can associate with up to a particular number of users simultaneously, and the number is limited by the number of orthogonal channels, $N_{CH}$. Thus, the following constraint (5) is used to limit the number of users that a BS can allow network access to at a time.

(5) $$A\times 1_{N_{U}\times 1}⪯N_{CH}\cdot 1_{N_{S}\times 1}$$

The federation-user mapping is also a binary relation as described in (6). That is, having the value of 1 for the (g,u)-the element in G indicates the federation *g* has decided to accept the user *u* so that the user can offload its processing load to the edge servers in the federation.

(6) $$G\in {\left\{0,1\right\}}^{N_{G}\times N_{U}}$$

In addition, one federation is exclusive of the rest by the assumption in this work, each user should become a member of a single federation by the constraint (7).

(7) $$G^{T}\times 1_{N_{G}\times 1}≃1_{N_{U}\times 1}$$

A user can offload its task to an ES if the user is assigned with an isolated virtual environment on the ES (8). The (s,u)-th element in Y corresponds to whether the edge server *s* has allowed user *u* to offload its task or not, if the value is 1 or 0, respectively.

(8) $$Y\in {\left\{0,1\right\}}^{N_{S}\times N_{U}}$$

In this study, we assume a containerized virtual environment such as Docker [40] which is light-weight and widely used in MEC [24]. Highly-loaded users may use multiple ESs for distributed task offloading, but the user can utilize only the ESs belonging to the same federation (9).

(9) $$Y^{T}⪯G^{T}\times I_{grp}$$

The portion of task offloaded to the device itself, one or more ESs and the cloud is determined by the corresponding variables $x_{user}$, $X_{es}$ and $x_{cloud}$, respectively (10).

(10) $$x_{user}\in {\left[0,1\right]}^{N_{U}\times 1},x_{cloud}\in {\left[0,1\right]}^{N_{U}\times 1},X_{es}\in {\left[0,1\right]}^{N_{S}\times N_{U}},$$

While the cloud is assumed to have enough resources to allow any amount of task offloading, both user devices and edge servers are of limited capacities as shown in (11) and (12), respectively.

(11) $$x_{user}⪯c_{user}$$

(12) $$X_{es}\times 1_{N_{U}\times 1}+h\cdot Y\times 1_{N_{U}\times 1}⪯c_{es}$$

Each *u*-th element and *s*-th element in $c_{user}$ and $c_{es}$ corresponds to the computing resource budget of user *u* and edge server *s*, respectively. To calculate the amount of resource in use for each BS, we also consider the overhead to run virtual containers, represented by *h*.

A user can offload its task to one or more ESs if there is a container dedicated to the user in the corresponding edge server as described in the constraint (13).

(13) $$X_{es}⪯Y$$

Each user’s QoS should be fully satisfied by the constraint (14), and it is assumed to be always possible due to the abundant resource in the cloud.

(14) $$x_{user}+x_{es}^{T}\times 1_{N_{S}\times 1}+x_{cloud}≃r$$

The last constraint (15) sets the upper-bound *b* for the workload offloaded to federations, which is used to achieve load balancing.

(15) $$\left(I_{grp}\times X_{es}\right)\times 1_{N_{U}\times 1}⪯b\cdot 1_{N_{G}\times 1}$$

Putting it all together, we have the following Stage-1 optimization problem (called P. 16).

$$\begin{array}{cc} (P.16)\min_{\begin{array}{c} A,G,Y,\\ x_{user},X_{es},\\ x_{cloud},b \end{array}}\;\; & \frac{\alpha }{s_{1}}b+\frac{\beta }{s_{2}}1_{1\times N_{U}}\times x_{cloud}+\frac{1-\alpha -\beta }{s_{3}}1_{1\times N_{U}}\times x_{user}\\ \mathrm{subject}\;\mathrm{to}\;\; & \left(2)-(15\right) \end{array}$$

Given the optimal solutions $A^{*}$, $G^{*}$ and $Y^{*}$ from the Stage-1 problem (P. 16), Stage-2 iteratively makes decisions on resource provisioning (i.e., VS and HS) and task offloading upon receiving real-time offloading requests. The Stage-2 optimization problem (called P. 17) is a subset of P. 16, and it is formally defined as below, where γ∈[0,1] is a design parameter, indicating the weight given to minimizing the use of the remote cloud resource.

$$\begin{array}{cc} (P.17)\min_{\begin{array}{c} x_{user},X_{es},\\ x_{cloud} \end{array}}\;\; & \frac{\gamma }{s_{2}}1_{1\times N_{U}}\times x_{cloud}+\frac{1-\gamma }{s_{3}}1_{1\times N_{U}}\times x_{user}\\ s.t.\;\;\; & \left(10),\;(11),\;(12),\;(13),\;(14\right) \end{array}$$

The proposed problem P. 16 is non-convex due to the binary (or integer in general) variables A and G. However, due to efficient algorithms such as branch-and-bound and branch-and-cut [41], the given problem can be efficiently solved by computer solvers such as CPLEX [42] and Gurobi [43]. Also, P. 16 is an off-line method that can run often, meaning that the computation complexity is of less importance. On the other hand, P. 17 is for a real-time, iterative algorithm that should run each time slot. Due to the linearity of problem P. 17, its time complexity is polynomial [44] and thus, it is applicable to be used as a real-time scheduling algorithm. In spite of the presence of binary variables (or integers, in general) in the proposed problem formulation, one can efficiently find the global optimal solution with the widely used computer solvers, such as Gurobi and CPLEX. For example, to solve mixed integer programming-type problems, Gurobi which is used in this work employs branch-and-bound and branch-and-cut methods which are widely used exact solutions [45].

## 4. Evaluation

In this section, we first present the parameters assumed and used in our evaluation, along with the layout of the BS/ES and users. Also, the various algorithms adopted for performance comparisons are enumerated. The simulation and evaluation is carried out on a high-performance workstation with an Intel Core i9 10940X CPU and 128 GB memory, and the reported values in this section are the average out of ten evaluations. The evaluations were carried out on two different network configurations, namely a grid network and random network presented in Section 4.1 and Section 4.2, respectively.

### 4.1. Even Distribution of Base Stations

**Figure 3:**
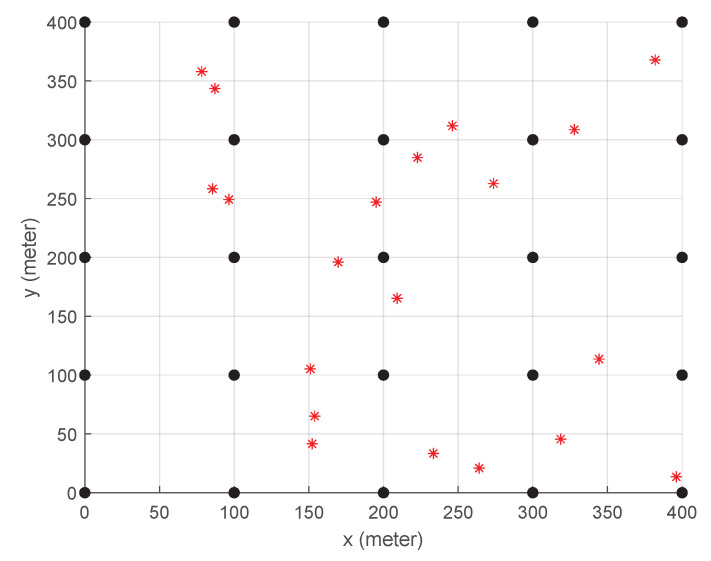
The layout of the assumed 400 m -by-400 m area where the 20 red stars and 25 black dots are the locations of users and BSs, respectively.

Figure 3 illustrates the assumed area for evaluation where 20 users (or user devices) and 25 BSs are evenly deployed. Users are placed at uniform random, while BSs are located at intersections on a grid. Each BS can communicate with users within 150 m radius coverage, and there are five orthogonal channels available so that up to five users are served simultaneously by a single BS. As shown in Figure 1, over the access network, there are 25 ESs federated into three groups and the ESs within the same federation are connected with high-speed communication links. We assume that the three federations of ESs are operated by different service providers, and thus HS, if supported, can occur with the ESs in the same federation but inter-federation HS is prohibited.

In our evaluation, we have abstracted both the users’ offloading requests and the computing capacities such that they are denoted by a unit-less number in the range of [0,1.0]. The rate at which the offloading request of each user is generated per unit of time is randomly chosen from Uniform[0.20,1.00]. The resource budget of each user and ES for each unit time is randomly drawn from Uniform[0.05,0.20] and Uniform[0.40,0.80], respectively, indicating the random background processing workload. The cloud is assumed to have large enough resources to handle any amount of request. A user’s request can be processed by the user’s device, one or more ESs in the same federation, and/or the cloud. To provide the offloading service to the user, the ES shall create a light-weight container which consumes h=0.05 amount of resource. The assumed parameters for evaluation are summarized in Table 1. The weights, α and β, are configured to 0.12 and 0.44, respectively, and the particular values are found by a heuristic approach.

We have implemented the NAFEOS and the simulation environment on MATLAB [46]. To solve the proposed optimization problem, we have used CVX [47] and Gurobi. For comparison, we have also implemented the following algorithms:

- NAFEOS: the optimal method proposed in this paper.
- RND (Random): random approach that makes decisions at random.
- RAG (Random Association and Grouping): same as NAFEOS, except that RAG randomly makes association and user-federation mapping (also called grouping).
- noHS (no Horizontal Scaling): same as NAFEOS, except that HS is not supported.
- noLB (no Load Balancing): same as NAFEOS, except that load balancing among federations is not supported.

**Figure 4:**
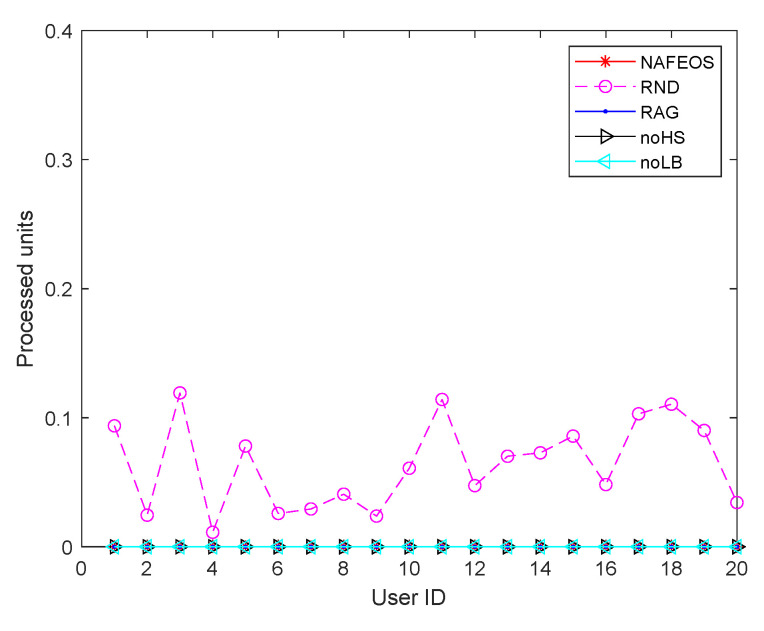
The amount of users’ requests processed locally at the users’ device on a grid network.

Figure 4 shows the amount of processing units that are handled locally at the users’ device. Except RND which randomly makes decisions, the rest of the algorithms perform optimal provisioning and task offloading. To be specific, NAFEOS, RAG, noHS, and noLB share a similar objective function that penalizes the use of users’ devices for processing. As a result, RND partially lets users process their own requests locally, whereas the other algorithms do not. The reason why it is penalized in this work is to save battery consumption on the users’ devices and to prolong their lifetime.

**Figure 5:**
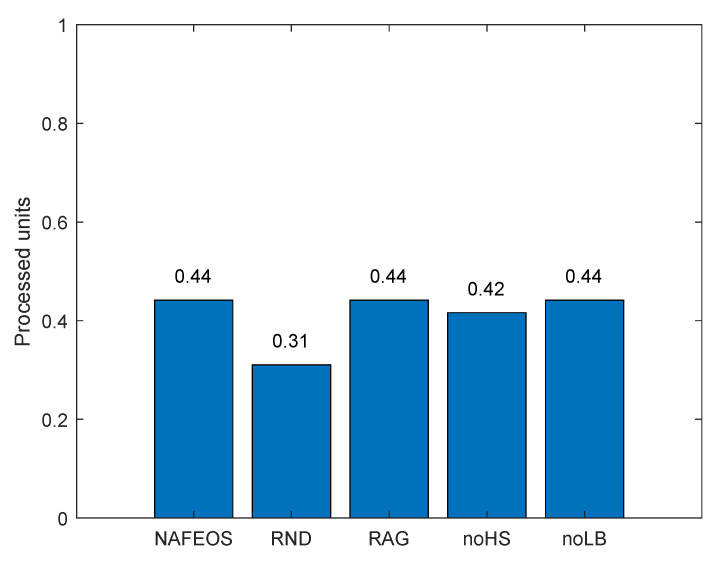
The average amount of users’ requests processed at the edge server on a grid network.

**Figure 6:**
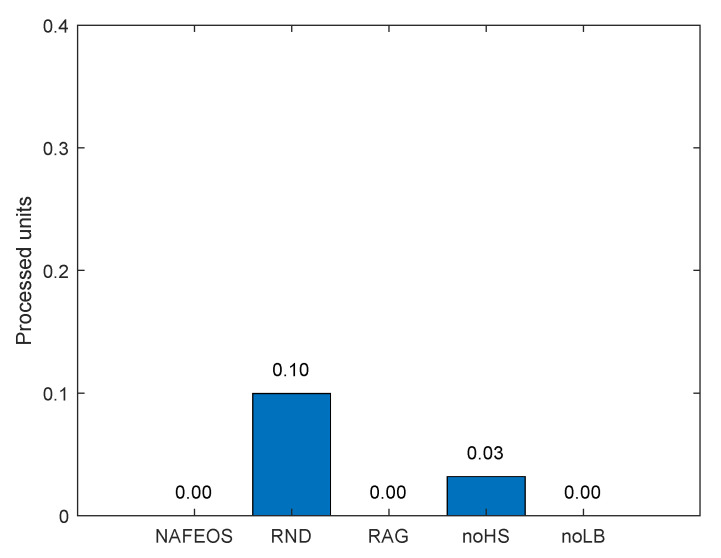
The average amount of users’ requests processed at the remote cloud data center on a grid network.

**Figure 7:**
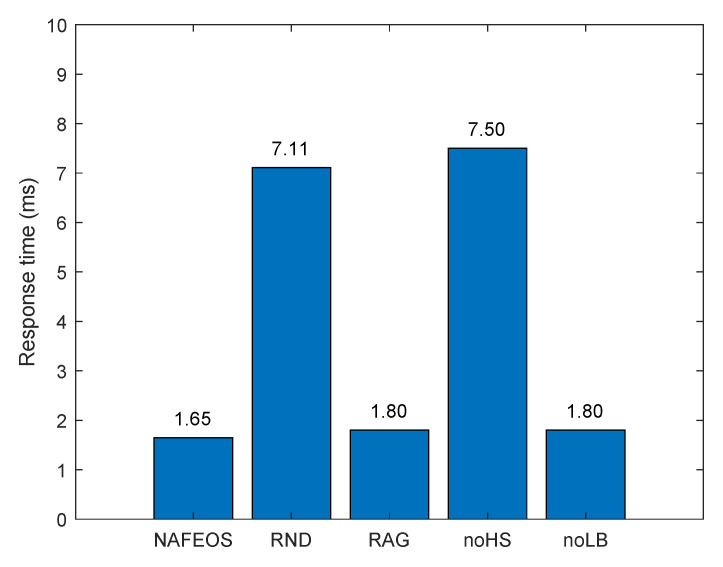
The average per-user offloading service response time ignoring the task processing time on a grid network.

Figure 5 and Figure 6 depict the average amount of processing units allocated at the ES and cloud, respectively, for each user’s task offloading. As it can be seen from both figures, the NAFEOS, RAG and noLB can provision the optimal amount of processing units by using HS, and thus, there is no offloading to the cloud. Although noHS is another optimal provisioning scheme, it does not perform HS. If a single ES is assigned to multiple users and their aggregate request exceeds the ES’s budget, the overflowing requests will be forwarded to the cloud. The downside of offloading to the cloud is the increased response time or end-to-end delay shown in Figure 7. With the assumption of a 5G cellular network as the underlying infrastructure [48], the achieved delay lends support to their relevance for various applications.

For our evaluation, edge-to-edge and edge-to-cloud delays are set to 1.5 ms and 15 ms, respectively [49]. Given the distance between each user and its associated BS which can be computed by using their locations, the propagation delay between the two can be computed by dividing the distance by the speed of light. As it is already discussed in Section 3.1, we assume that the BS-user delay is negligibly small compared to the rest, edge-to-edge delay within the same federation is small, and edge-to-cloud delay is the largest in this study. Since the transmission range of a BS is up to 150 m, dividing the worst-case user-BS distance by the speed of light (i.e., 3 × 10${}^{8}$ m/s) yields 50 µs, which is much smaller than the edge-to-edge delay. To be specific, the response time is computed as follows. For each time slot, for the proposed NAFEOS and other approaches that are considered for comparison, solve the corresponding algorithm to make decisions on self-offloading, ES offloading, and cloud offloading. Once the decision is carried out, the response time can be computed. Let $d_{BS}$, $d_{e2e}$ and $d_{e2c}$ be the one-way delay for BS-user, edge-to-edge, and edge-to-cloud. Then, for the following cases, the response time which excludes the time taken to process the offloaded task is computed as below.

- self-offloading yields zero response time
- offloading to ES without HS yields $2d_{BS}$
- offloading to ES with HS yields $2(d_{BS}+d_{e2e})$
- offloading to the cloud yields $2(d_{BS}+d_{e2c})$
- offloading to ES without HS and to the cloud yields $2(d_{BS}+d_{e2c})$
- offloading to ES with HS and to the cloud yields $2(d_{BS}+d_{e2e}+d_{e2c})$.

It is worth mentioning that the reported response time in this section is per-user average value, meaning that the summation of all response times is divided by the number of users reported in this section.

By using the above delay configurations, we can compute the average per-user response time (i.e., twice the end-to-end delay) as shown in Figure 7.

As it can be seen from the figure, the three optimal methods, i.e., NAFEOS, RAG, and noLB, outperformed the rest. The main reason for such low response time is because they do not offload to the cloud, which is causing the largest delay. In addition, due to the balanced load among federations, it is less likely that a certain ES is highly overloaded for the NAFEOS scheme. As a result, HS, which is causing an additional delay for the transmission among ES, is also minimized. Thus, the NAFEOS scheme achieved the lowest response time, although the improvement compared to RAG and noLB is insignificant. Both RND and noHS offload users’ requests to the remote cloud which increased the response time significantly. Among the two, RND achieved slightly better performance because it randomly lets users process their own tasks which is causing zero network delay.

**Figure 8:**
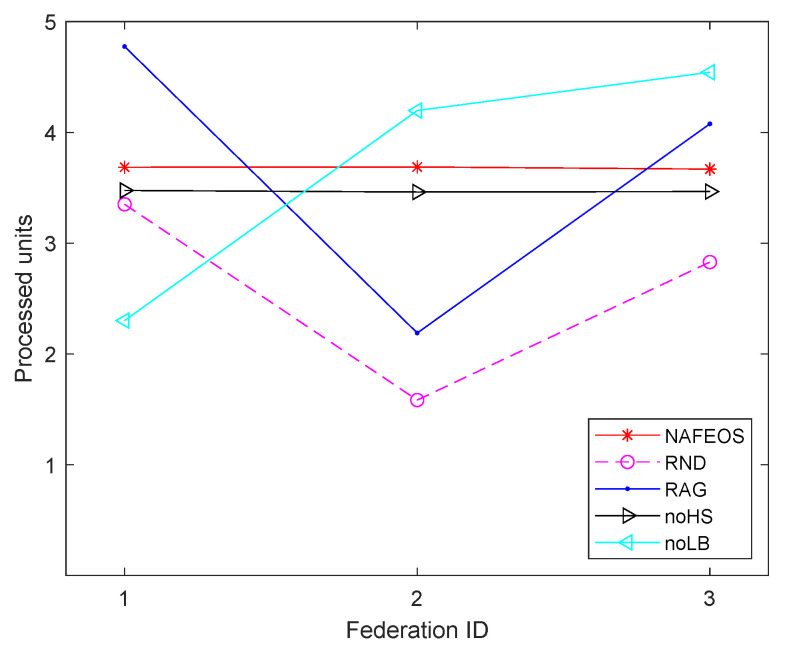
The average amount of processed units at each federation on a grid network.

**Figure 9:**
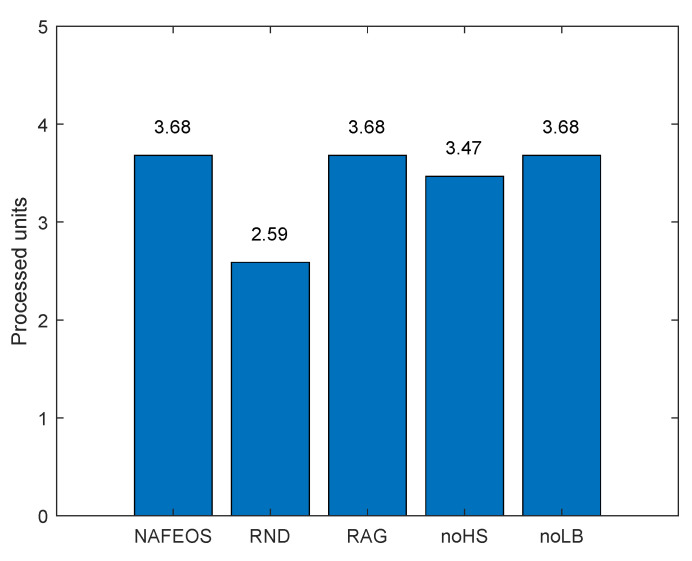
The average amount of processed units per federation on a grid network.

**Figure 10:**
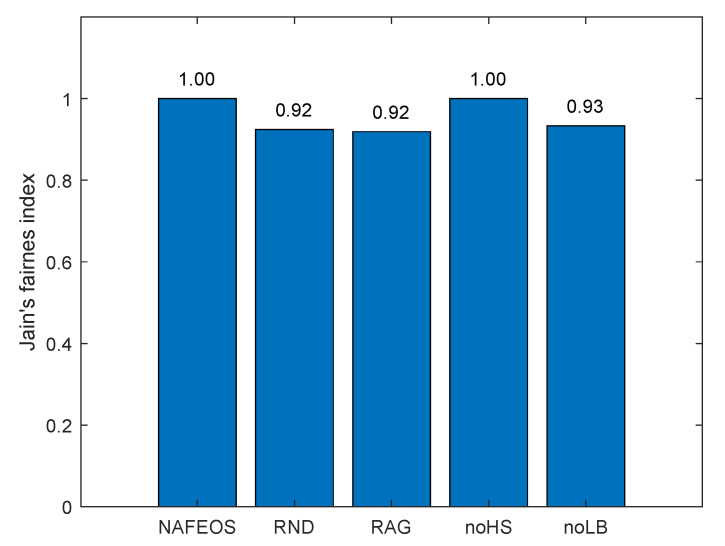
The performance of the fair distribution of the processed units among federations on a grid network.

The following figures, Figure 8, Figure 9 and Figure 10, show the load-balancing performance among the three federations. The three optimal schemes, i.e., NAFEOS, RAG, and noLB, offload the entire task to ESs, and thus they achieve the largest offloaded units as shown in Figure 9 on average. However, due to the ill-considered association/federating and missing load-balancing features in RAG and noLB, respectively, their fairness performances are degraded as shown in Figure 10. To measure the load-balancing performance among federations, we have used the widely used Jain’s fairness index [50] which measures the fairness as follows:

$$J\left(x_{1},x_{2},\cdots ,x_{n}\right)=\frac{{\left(\sum_{i=1}^{n}x_{i}\right)}^{2}}{n\cdot \sum_{i=1}^{n}x_{i}^{2}}.$$

Although noHS achieved almost perfect fairness, due to the inability to perform HS, it has offloaded a certain amount of task to the cloud, yielding lower performance than NAFEOS as shown in both Figure 8 and Figure 9. Due to the random deployment of users, RND was able to achieve high fairness performance, but still it is outperformed by the NAFEOS.

### 4.2. Random Distribution of Base Stations with Higher Task Generation Rate

**Figure 11:**
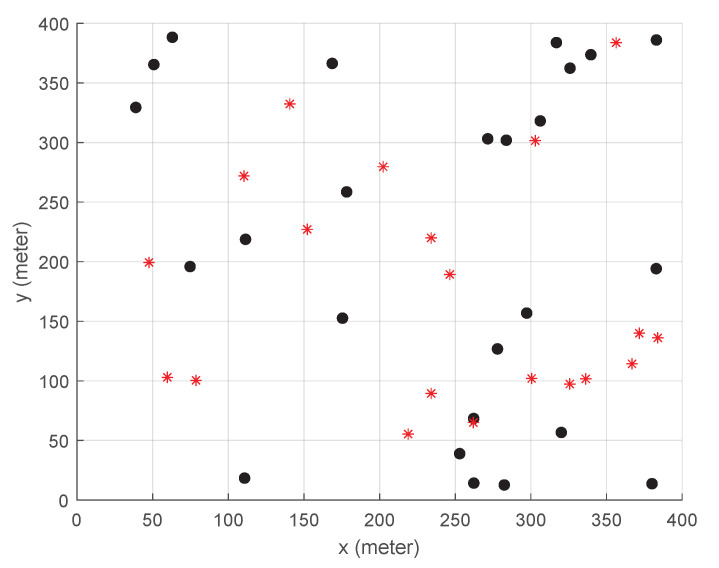
The layout of the assumed 400 m-by-400 m area where the 20 red stars and 25 black dots are the locations of users and BSs, respectively, that are randomly distributed.

An additional evaluation has been carried out on a randomly located BS (see Figure 11) with a higher task generation rate. Table 2 summarizes only the parameters that are different from the previous ones in Table 1, and the weight parameters are heuristically chosen as before.

**Figure 12:**
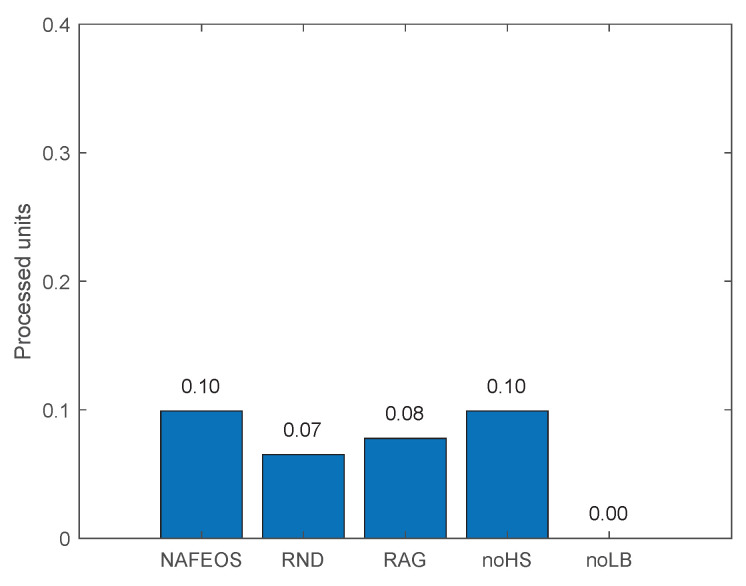
The amount of users’ requests processed locally at the users’ device on a random network.

Figure 12 shows the amount of task processed by the device itself. Except for noLB, all approaches let users process a small amount of load by themselves. This is quite different from the results from the evenly distributed BSs scenario with a moderate task generation rate in Figure 4. When each user is surrounded by a number of ESs and the amount of load to process is moderate, users do not offload tasks to themselves to minimize their battery consumption as shown in Figure 4. However, on the assumed network in this section where ESs are randomly deployed and the load generation rate is high, some users may not secure enough resources on ESs, and thus to reduce the service delay, devices process a small mount of tasks by themselves. On the other hand, noLB does not have restrictions on the even distribution of the load among federations and thus, it utilizes as much resource on ES as possible, resulting in no self-offloading on average.

**Figure 13:**
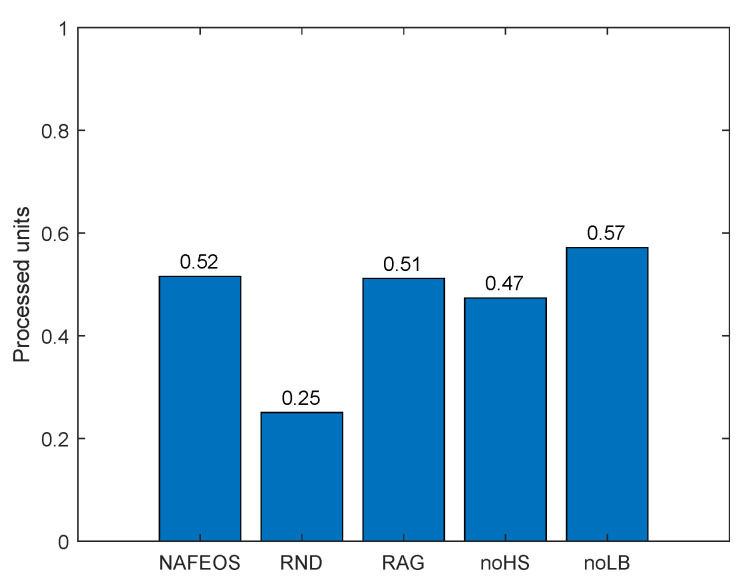
The average amount of users’ request processed at edge server on a random network.

**Figure 14:**
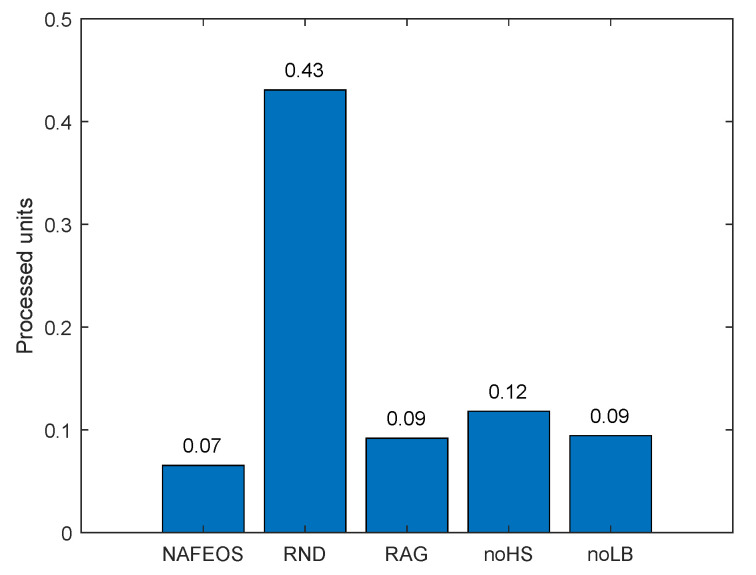
The average amount of users’ request processed at the remote cloud data center on a random network.

**Figure 15:**
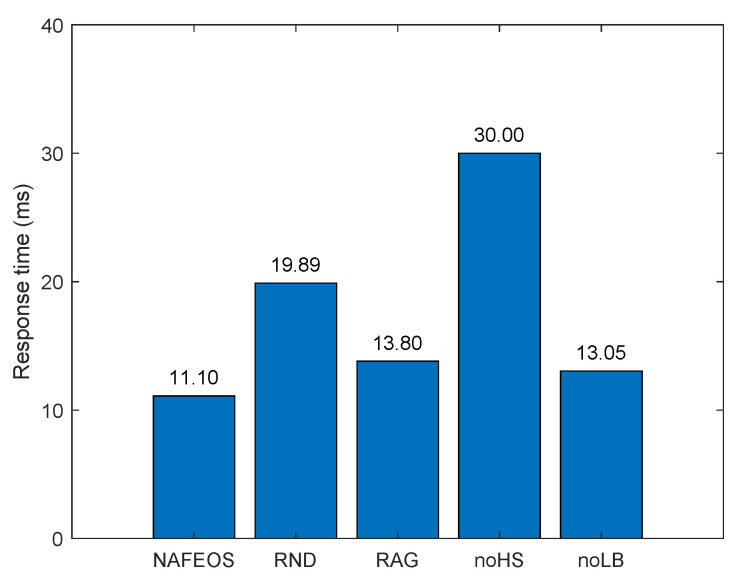
The average per-user offloading service response time ignoring the task processing time on a random network.

Figure 13 and Figure 14 show the amount of tasks offloaded to the ES and cloud, respectively. Except RND which randomly offloads the load, the rest of the approaches utilize a lot of resources from ES and a few from the cloud. The main reason for this is that utilizing ES yields a shorter delay. However, due to the limited resources on the ES, a small portion of the load is processed on the cloud anyway. The proposed NAFEOS offloads the least amount of load to the cloud, which effectively reduces the response time as shown in Figure 15. One interesting result here is that although RND offloads more to the cloud compared to noHS, its response time is much less than noHS. After analysis, what we found is as follows. In RND, a small number of users offloaded a lot of load to the cloud. On the other hand, noHS lets many users offload a small amount of task to the cloud. The noHS is not allowed to perform horizontal scaling. Thus, once the assigned ES is operating at its full capacity, users redirect the remaining load to the cloud.

**Figure 16:**
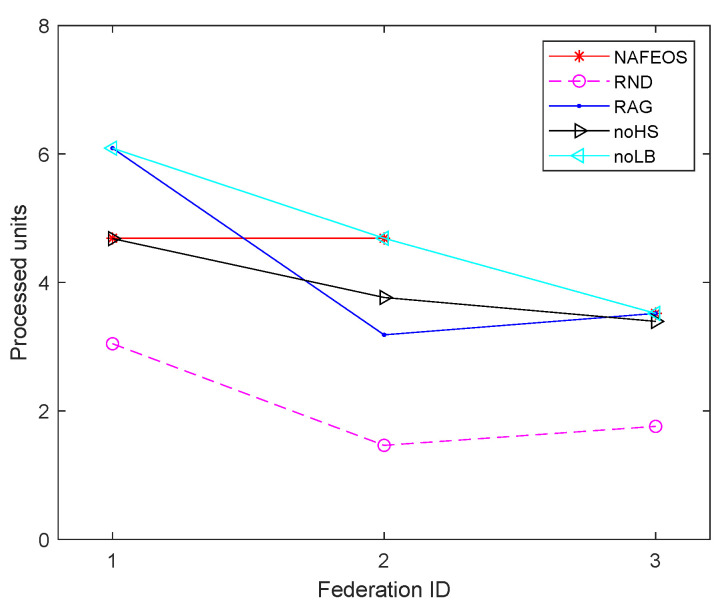
The average amount of processed units at each federation on a random network.

**Figure 17:**
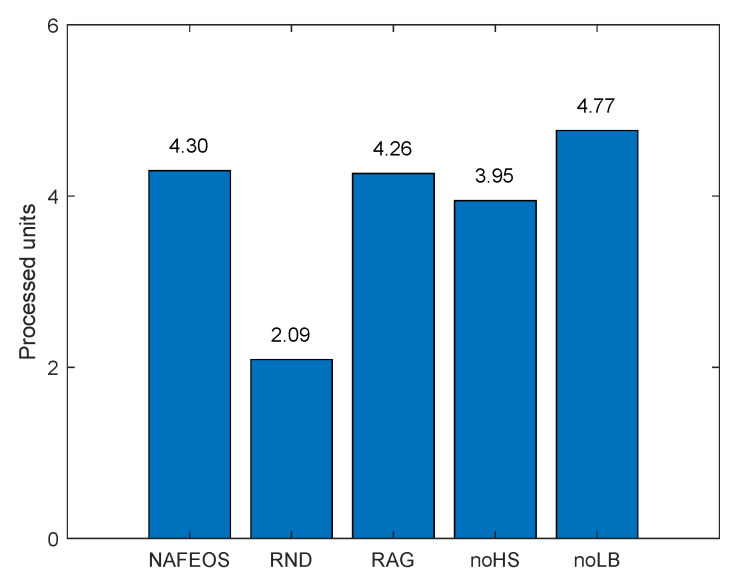
The average amount of processed units per federation on a random network.

**Figure 18:**
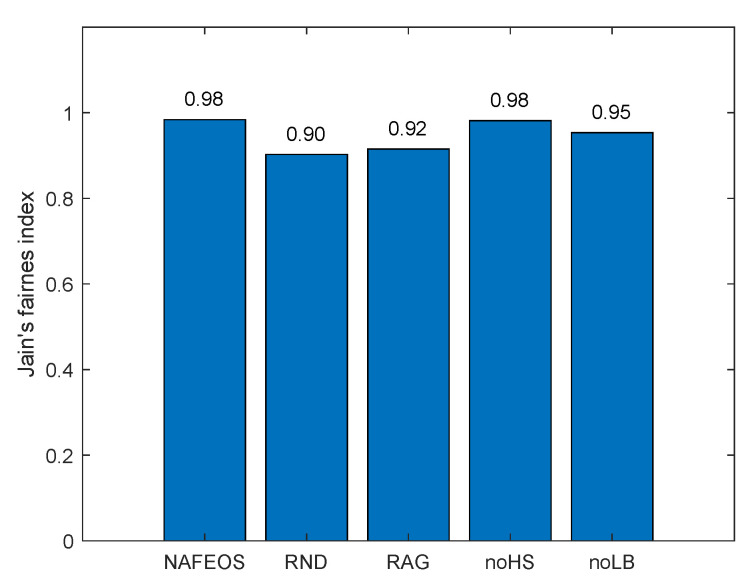
The performance of the fair distribution of the processed units among federations on a random network.

Figure 16, Figure 17 and Figure 18 show the load balancing-related performance among federations. From both Figure 16 and Figure 18, it is clear that the proposed NAFEOS achieves the highest load-balancing performance. Although noHS has achieved comparable fairness performance to NAFEOS, the amount of load offloaded to ESs is less than that of NAFEOS as shown in Figure 17. That is, the proposed NAFEOS can not only achieve high load-balancing performance, but also utilize as many resources on ES as possible which is an effective approach to reduce both the service delay and the battery consumption at the user device. Although noLB maximizes the use of ES resources, due to the lack of the load-balancing feature, its fairness performance is lower than that of NAFEOS.

## 5. Conclusions

In this paper, we have introduced NAFEOS, an approach for optimal resource scheduling and task distribution in edge computing. In the NAFEOS system architecture, edge servers are federated, forming exclusive sets of available edge servers. A user’s association with a base station determines which edge server and federation the user can access. Additionally, task offloading for users can be facilitated through horizontal and vertical scaling to meet the users’ Quality of Service (QoS) requirements.

NAFEOS implements a two-stage approach, where the first Stage-1 solves the long-term decisions on the association between base station and user and the federation assignment between edge server federation and user along with initial provisioning of edge server resources. The following Stage-2 algorithm is repeatedly invoked on short time scales (or time slots) to make optimal decisions on edge server resource provisions by means of vertical and horizontal scaling. Then, it distributes the users’ offloading requests to different layers.

The performance of NAFEOS, as well as several comparison algorithms, was systematically evaluated in two distinct network scenarios. The first scenario involved an even distribution of base stations (BS), while the second scenario involved a random distribution of BS with a higher task generation rate. In a grid network characterized by uniform distribution of BS and users, NAFEOS demonstrates outstanding performance in processing user requests at the ES. NAFEOS achieves a processing rate of 0.44 processing units, outperforming RND, RAG, noHS, and noLB. NAFEOS efficiently allocates processing units to the cloud, thus reducing the demand for offloading and minimizing cloud utilization. It demonstrates optimal provisioning with a significantly lower per-user offloading service response time of 1.65 ms. NAFEOS shows its outstanding efficiency by achieving a processing unit count of 3.68 per federation. NAFEOS’ outstanding load balancing and fairness among federations, confirmed by Jain’s fairness index, establish NAFEOS as the optimal choice. In a random network with a higher task generation rate, NAFEOS shows outstanding results in task processing across different components. Specifically, it achieves a processing capacity of 0.10 units at users’ devices, 0.52 units at the edge server, and effectively minimizes cloud offloading with a processing capacity of 0.07 units. The observed results show a lower average per-user offloading service response, with a value of 11.10 ms compared to other algorithms. NAFEOS also shows outstanding load-balancing capabilities, outperforming other algorithms with an average of 0.98 processed units per federation. The evaluation results show that NAFEOS is an effective approach for enhancing the efficiency and overall performance of IoT networks through association and federation, horizontal and vertical scaling, and workload balancing among federations.

NAFEOS has the potential to enhance network efficiency, extend user device lifetimes, and reduce response times in various low-powered Internet of Things applications such as remote monitoring of assets, smart surveillance systems, predictive maintenance in manufacturing, content caching and monitoring, and smart city/home. In particular, in the real-world applications operated mainly by low-power devices with network resource limits, the proposed NAFEOS is expected to provide the best efficiency gain. While the specific benefits may vary, the underlying principles of resource optimization remain consistent. In future work, we plan to carry out an empirical evaluation and integrate the federated learning into NAFEOS, further enhancing its ability to process user requests efficiently.

## Figures and Tables

**Table 1:** Parameters used for evaluation on a grid network.

Parameter	Value
Number of users	20, distributed randomly
Number of BS/ES	25, distributed evenly
BS transmission range	150 m
Number of orthogonal channels	5
Number of federations	3
Offloading request rate	Uniform[0.20,1.00] per user
Resource budget	Uniform[0.05,0.20] per user
	Uniform[0.40,0.80] per ES
*h*	0.05 (container operating overhead)
weights	α=0.12,β=0.44

**Table 2:** Changed parameters for evaluation on a random network.

Parameter	Value
Number of BS/ES	25, randomly distributed
Offloading request rate	Uniform[0.60,1.00] per user
weights	α=0.22,β=0.48

## Data Availability

The data presented in this study are available on request from the corresponding author.
